# Integrated analysis of single-cell RNA-seq and bulk RNA-seq reveals RNA N6-methyladenosine modification associated with prognosis and drug resistance in acute myeloid leukemia

**DOI:** 10.3389/fimmu.2023.1281687

**Published:** 2023-10-31

**Authors:** Zhongzheng Li, Xin Liu, Lan Wang, Huabin Zhao, Shenghui Wang, Guoying Yu, Depei Wu, Jianhong Chu, Jingjing Han

**Affiliations:** ^1^ State Key Laboratory of Cell Differentiation and Regulation, Henan International Joint Laboratory of Pulmonary Fibrosis, Henan Center for Outstanding Overseas Scientists of Pulmonary Fibrosis, College of Life Science, Institute of Biomedical Science, Henan Normal University, Xinxiang, China; ^2^ Institute of Blood and Marrow Transplantation, Collaborative Innovation Center of Hematology, Soochow University, Suzhou, China; ^3^ The First Affiliated Hospital of Soochow University, National Clinical Research Center for Hematologic Diseases, Jiangsu Institute of Hematology, Collaborative Innovation Center of Hematology, Soochow University, Suzhou, China

**Keywords:** acute myeloid leukemia, N6-methyladenosine modification, subtype, single-cell transcriptome, tumor microenvironment

## Abstract

**Introduction:**

Acute myeloid leukemia (AML) is a type of blood cancer that is identified by the unrestricted growth of immature myeloid cells within the bone marrow. Despite therapeutic advances, AML prognosis remains highly variable, and there is a lack of biomarkers for customizing treatment. RNA N6-methyladenosine (m^6^A) modification is a reversible and dynamic process that plays a critical role in cancer progression and drug resistance.

**Methods:**

To investigate the m^6^A modification patterns in AML and their potential clinical significance, we used the AUCell method to describe the m^6^A modification activity of cells in AML patients based on 23 m^6^A modification enzymes and further integrated with bulk RNA-seq data.

**Results:**

We found that m^6^A modification was more effective in leukemic cells than in immune cells and induced significant changes in gene expression in leukemic cells rather than immune cells. Furthermore, network analysis revealed a correlation between transcription factor activation and the m^6^A modification status in leukemia cells, while active m^6^A-modified immune cells exhibited a higher interaction density in their gene regulatory networks. Hierarchical clustering based on m^6^A-related genes identified three distinct AML subtypes. The immune dysregulation subtype, characterized by *RUNX1* mutation and *KMT2A* copy number variation, was associated with a worse prognosis and exhibited a specific gene expression pattern with high expression level of *IGF2BP3* and *FMR1*, and low expression level of *ELAVL1* and *YTHDF2*. Notably, patients with the immune dysregulation subtype were sensitive to immunotherapy and chemotherapy.

**Discussion:**

Collectively, our findings suggest that m^6^A modification could be a potential therapeutic target for AML, and the identified subtypes could guide personalized therapy.

## 1 Introduction

Acute myeloid leukemia (AML) represents an infrequent hematologic malignancy distinguished by the aberrant expansion of precursor cells in the myeloid lineage, resulting in a perturbed differentiation process. In recent decades, allogeneic hematopoietic stem cell transplantation (allo-HSCT) has emerged as a pivotal therapeutic intervention, substantially enhancing the overall survival of eligible AML patients (1). However, the effectiveness of AML treatment and the issue of relapse continue to pose significant clinical challenges. Until recently, there has been a considerable gap in meeting the treatment needs of AML patients.

RNA methylation, particularly N6-methyladenosine (m^6^A) methylation, constitutes the prevalent intramolecular modification observed within eukaryotic mRNA molecules. Such modification is involved in regulating various aspects of RNA, including splicing, stability, localization, and translation (2, 3). In recent times, m^6^A modifications have not solely surfaced as a novel stratum of epigenetic control within the realm of cancer; they have also exhibited substantial therapeutic promise for addressing diverse forms of malignancies (3–6). Notably, current studies have highlighted the potential of m^6^A modifications in elucidating the pathogenesis and therapeutic aspects of AML. Specifically, the m^6^A methyltransferases *METTL3* and *METTL14* can control and/or maintain myeloid leukemia cells (7–9). The m^6^A readers *IGF2BP2* and *IGF2BP3* promote AML development in an m^6^A-dependent behavior by controlling the expression level of critical genes in the glutamine metabolism pathways (10, 11). The RNA-binding protein YBX1 plays a pivotal role in sustaining myeloid leukemia cells through an m^6^A-dependent mechanism, wherein it regulates the stability of *BCL* (12). *FTO* plays an oncogenic role in AML as an m^6^A RNA demethylase. Small molecule inhibitors that target *FTO* have the potential to be used in the treatment of AML (13, 14). These findings provide valuable insights into the crucial and intricate involvement of m^6^A modification and its regulators in AML, highlighting a promising avenue for AML treatment.

In this study, we identified two distinct patterns of m^6^A modification intensity based on the m^6^A activity score. We found that high-intensity modes significantly impact the prognosis of AML. Additionally, we conducted a comprehensive analysis of the role of m^6^A modification in AML subtypes, including their genomic alterations, tumor immune microenvironment, and immunotherapy implications. This examination notably enriches our comprehension of the molecular processes that contribute to m^6^A modification’s role in the development of AML and its impact on the effectiveness of drug treatments. Overall, our findings provide a solid foundation for the development of m^6^A modification-targeting therapies for AML and suggest that this approach could be an effective and specific strategy for cancer treatment.

## 2 Materials and methods

### 2.1 Data download and processing

We got patient data for acute myeloid leukemia (AML) from the TCGA (The Cancer Genome Atlas) database, which was downloaded from the following source: https://xena.ucsc.edu/. RNAseq data from a total of 151 samples were re-analyzed. Clinical survival information was obtained for 132 patients and somatic mutation information was obtained for 143 patients. To verify, we conducted searches in the GEO database using the keywords ‘acute myelogenous leukemia’ and ‘LAML’ to identify relevant datasets. Specifically, we included datasets in our analysis if they met the following criteria: (1) Adequate sample size (n > 200). (2) Using RNA-seq instead of microarrays. (3) Count matrix is provided for further analysis. GSE106291 (n = 250) and GSE146173 (n = 246) were selected to further analysis (15, 16). We utilized the dataset GSE178926, which comprises targeted immune gene expression profiles derived from pre-treatment bone marrow samples of acute myeloid leukemia patients undergoing treatment with pembrolizumab and the hypomethylating agent azacytidine (ClinicalTrials.gov Identifier: NCT02845297) as described by Rutella et al. in 2022 (17), for the purpose of immunotherapy prediction. Single-cell RNA sequencing (scRNA-seq) data from 12 patients diagnosed with acute myeloid leukemia (AML) were obtained from the dataset provided by van Galen et al. (18).

### 2.2 Re-analysis of scRNA-seq

The single-cell RNA sequencing (scRNA-seq) count data underwent normalization using the ‘Seurat’ R package (19), followed by log-transformation with an offset value of 1 and subsequent scaling. We discerned genes displaying significant variability through the utilization of the ‘FindVariableFeatures’ function, wherein the ‘vst.method’ parameter was configured as ‘vst’. We conducted Principal Component Analysis (PCA) on the highly variable genes, utilizing the top 30 principal components for subsequent analyses. Cell type annotation was obtained from Zeng et al. (20). In order to represent the outcomes visually, we employed t-distributed stochastic neighbor embedding (t-SNE) to reduce the complexity of the dataset. The RunTSNE function was used to generate a 2-dimensional t-SNE plot based on the top 30 principal components. The resulting t-SNE plot was then used to visualize the clustering results. Subsequently, we employed the ‘FindAllMarkers’ function to identify cluster-specific markers, with the ‘method’ parameter set to ‘MAST’ (21).

### 2.3 m^6^A modification activity score

We obtained 23 m^6^A modification enzymes (*FTO, CBLL1, FMR1, HNRNPC, HNRNPA2B, IGF2BP1, IGF2BP2, ELAVL1, IGF2BP3, ALKBH5, LRPPRC, METTL3, METTL14, RBM15, RBM15B, VIRMA, WTAP, YTHDF1, YTHDF2, YTHDC1, YTHDC2, YTHDF3, ZC3H13*) and scored their m^6^A modification activity using the AUCell R package (22). The enzymes were used as a gene set to calculate the area under the curve (AUC) value. This value was used to rank gene expression for each cell, reflecting the proportion of highly expressed genes in the gene set for that cell. To identify the active gene set, we used the “AUCell_exploreThresholds” function to calculate the threshold value (0.048). Using the AUC scores of each cell, we colored the cell clustering UMAP embedding to show which cells were active.

### 2.4 Regulon analysis and signature enrichment

To assess the activity of transcription factor (TF) regulons in the context of single-cell RNA sequencing (scRNA-seq) data, we conducted a regulon analysis employing the SCENIC framework. Following the guidelines outlined by Van de Sande et al., we leveraged the Docker image of pySCENIC and utilized logarithmically transformed expression counts obtained from AML cells for input data (23). The identification of putative transcription factors (TFs) was carried out with default parameters and a compiled list of human TFs from Lambert et al. (24). To refine potential TF-target interactions within each regulon, we incorporated CisTarget, which entailed the utilization of established human TF motifs databases annotated at intervals of 500 base pairs, 5 kilobases (kb), and 10 kb from transcriptional start sites. Additionally, a drop-out masking strategy was applied during this refinement process. Subsequently, the enrichment of these refined TF regulons was assessed using AUCell, and enrichment scores were scaled for visualization purposes.

### 2.5 GRN construction

We generated gene regulatory networks (GRNs) for both m^6^A-active and m^6^A-inactive cell populations using the R package bigSCale2 (25, 26). To do so, we first separated the m^6^A-active and m^6^A-inactive cells and generated a cell count matrix for each m^6^A modification state using Seurat’s GetAssayData function. We then filtered the resulting matrices to remove genes with ensemble identifiers and passed them to bigSCale2 to construct the networks. The networks were generated under the “normal” clustering parameter, with an edge cutoff set to the top 0.9 quantile for correlation coefficient. We visualized the networks using the R package igraph, and each network’s layout was derived from the Fruchterman-Reingold algorithm.

### 2.6 Construction of cell-specificity m^6^A related signature

The single-cell approach enables a detailed exploration of the m^6^A modification model. We employed a likelihood-ratio test to establish a m^6^A-related gene signature for each cell-type-specific cluster. This entailed identifying differentially expressed genes between cells exhibiting m^6^A activity and those that are m^6^A inactive within each cluster. We focused on identifying m^6^A related genes based on the size of effect value (log2FC). The m^6^A-related genes within the various cell types were identified using the following criteria: |log2FC| > 0.25, percentage of cells (pct) < 0.1, and p-value < 0.05. We prefer to obtain m^6^A-related genes specific to each cell type, we therefore used and evaluated the cell specificity of m^6^A-related genes. Cell-specific genes within the different cell types were identified using the following criteria: log2FC > 0.25, percentage of cells (pct) < 0.1, and p-value < 0.01. Cell-specificity m^6^A related signature emphasizes strict cell specificity and comprehensive m^6^A modification profile, we thus relaxed the screening criteria for m^6^A-related genes and limited the screening criteria for cell markers.

### 2.7 Reanalysis of bulk RNA-seq

DEseq2 software was used to detect Differentially expressed genes (DEGs). Count matrix was scaled by variance-stabilizing transformation. We identified differentially expressed genes (DEGs) among the three subtypes using a threshold of |log2FC| ≥ 1 and a false discovery rate (FDR) ≤ 0.01. Subsequently, we conducted Gene Ontology (GO) analysis and Kyoto Encyclopedia of Genes and Genomes (KEGG) analysis, visualizing the results through Metascape. Furthermore, Molecular Signatures Database V7.4 of hallmark gene sets were used for GSEA by the R package ClusterProfiler.

### 2.8 Clustering, ESTIMATE, and ssGSEA

For TCGA-LAML, we integrated m^6^A related genes from five types of blood cancer cells and further identified the genes that determine survival status by Cox regression. We conducted hierarchical clustering analysis using the ‘ConsensusClusterPlus’ package (R implementation, K = 3) based on the 40-genes (Cox regression p-value < 0.05) from cell-specificity m^6^A Related Signature. We utilized ESTIMATE to assess the stromal score, immune score, ESTIMATE score, and tumor purity for each LAML sample (27). The ssGSEA was conducted to determine the enrichment levels of immune-related pathways in each sample.

### 2.9 Comparing tumor immune microenvironment

We conducted a systematic ssGSEA analysis to assess the activation levels of 17 immune pathways, as defined by ImmPort (28). Additionally, we quantified the expression levels of 78 immunomodulators based on a prior study, with the aim of exploring the immune microenvironment in LAML. To discern differences in immune signatures, including immune pathways and immunomodulators, among LAML subtypes, we employed both the Kruskal-Wallis test and the Wilcoxon rank-sum test.

### 2.10 Survival analysis

We defined overall survival (OS) as the duration between diagnosis and either death or last follow-up. We evaluated the differences in OS within subtypes in each cohort using Mantel-Cox log-rank tests, implemented using the R package ‘survival’. Kaplan-Meier plots were generated using the R package ‘survminer’ to visualize the survival curves for each cluster. We also derived univariate and pairwise hazard ratios (HRs) for each cluster using Cox proportional hazards regression.

### 2.11 Construction of composite machine learning model

The genes that satisfy the two conditions are used to build the machine learning (ML) model: 1. m^6^A related genes; 2. FDR<0.01 in the difference analysis of the three subtypes. We employed a systematic machine learning (ML)-based framework to construct subtype prediction models. The process involved several key steps: 1. Data Preprocessing: This step encompassed imputing missing values and scaling continuous features. Continuous features were standardized to have a zero mean and unit variance. 2. Data Splitting: Following preprocessing, we divided the data into two sets, a training dataset for model development, and a validation dataset for assessing model accuracy. The data split was random and maintained a ratio of 7:3. 3. Model Development: For each of the five ML algorithms (random forests, support vector machines, naïve Bayes, K-nearest neighbors, and neural networks), we developed optimal models using the training dataset. 4. Validation: To ensure robustness, we required consistent results from at least three models for each sample. All of the aforementioned modeling steps were implemented using R (version: 4.1.2).

### 2.12 Genomic analysis

We conducted Copy Number Alteration (CNA) analysis using GISTIC2.0 on the TCGA LAML cohort. We examined variations in amplification or deletion events at the gene level across the three subtypes. To visualize the Copy Number Variation (CNV) data, we utilized the ‘ComplexHeatmap’ package in R, creating a waterfall plot. Additionally, we calculated the Tumor Mutation Burden (TMB) by determining the number of mutations per patient.

### 2.13 Immunotherapy response score generation

Immunotherapy signature feature selection was performed using differentially expressed genes between CR (Complete Remission) and NR (Non-Responders) samples in the GSE178926 dataset. Sample identity information was established based on Rutella et al. study (17). We selected genes based on their association with CR compared to NR samples, utilizing Elastic Net penalized logistic regression (glmft algorithm, glmnet package) with alpha = 0.6 and lambda = 0.06 to accommodate the high degree of correlation observed among some genes. We opted for Leave-One-Out Cross-Validation (LOOCV) to maximize our training set for validation, especially given our small dataset size (n = 33), as LOOCV exhibits low bias.

### 2.14 Chemotherapeutic response prediction

Chemotherapeutic response predictive model is based on the Cancer Genome Project (CGP) data from the Genomics of Drug Sensitivity in Cancer (GDSC) project, using gene expression and drug sensitivity data from cancer cell lines. The R package “pRRophetic” utilized ridge regression to estimate the half-maximal inhibitory concentration (IC50) for each sample (29). Model training was performed using blood-type cell lines through 10-fold cross-validation based on the GDSC’s training set. A total of 45 cell lines derived from hematological cancers were utilized. To mitigate batch effects, we employed the ‘combat’ algorithm with default settings and considered only tissue types labeled as ‘blood’ Duplicate gene expression data were averaged.

### 2.15 Statistical analyses

The statistical analyses were conducted utilizing R software, version 4.1.2. The comparison of two groups was established via Wilcoxon-rank sum test, while analysis of more than three groups was accomplished through execution of Kruskal-Wallis test. The benchmark for significance was established with p-value, wherein a level of 0.05 or lower was deemed significant, while a level of 0.01 or lower was deemed extremely significant.

## 3 Results

### 3.1 Reanalysis of scRNA-seq data identifies active and inactive m^6^A methylation cells in AML

**Figure 1:**
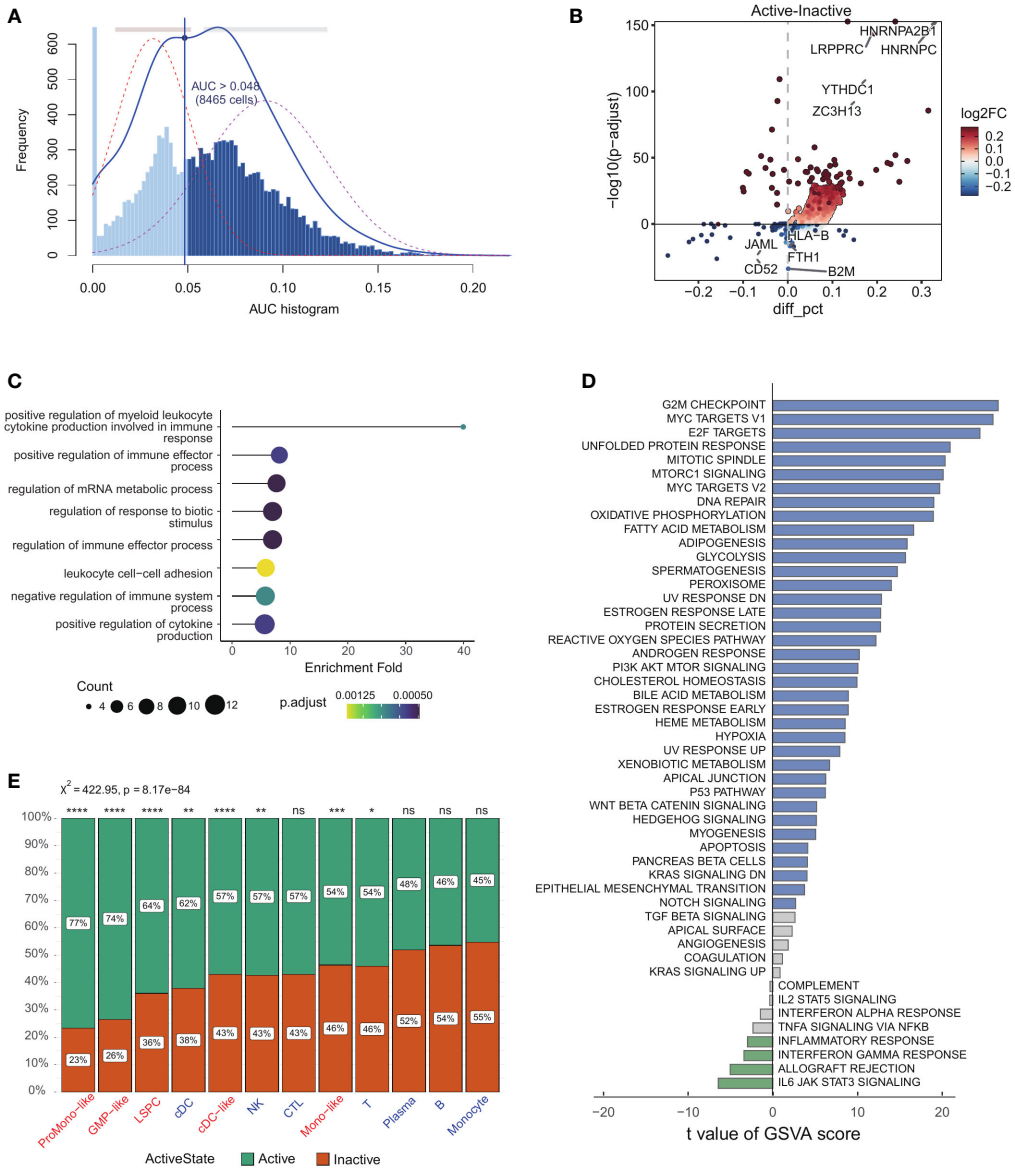
Identification of active and inactive m^6^A methylation cells in AML. **(A)** Score of 23 m^6^A modification activity. The threshold was chosen as 0.048 and the m^6^A modification score of 8,465 cells exceeded the threshold value (The dotted lines overlaying each distribution represent Gaussian fits to the distribution data.). **(B)** The volcano plot illustrates the differential expression of genes between m^6^A modification-active cells and m^6^A modification-inactive cells. **(C)** GO gene set enrichment analysis results were shown as lollipop plot where on x-axis, -log of FDR adjusted p-value for GO terms were shown. **(D)** Barplot of significantly activated pathways in the m^6^A-active (blue) and m^6^A-inactive (green) cells. **(E)** Stacked barplots showing the frequencies of m^6^A-active and m^6^A-inactive cells in 14 cell types (p-value > 0.05: ns, p-value < 0.05: *, 0.05 < p-value < 0.01:**, 0.01 < p-value < 0.001:***, 0.001 < p-value < 0.0001:****).

we conducted a comprehensive reanalysis of scRNA-seq data obtained from 13,653 cells from 12 patients diagnosed with AML (GSE116256) (18, 20). Cell identity information was established based on prior studies encompassing five leukemic cell types (LSPC, ProMono-like, Mono-like, GMP-like and cDC-like) and seven non-leukemic immune cell types (B, T, natural killer, plasma, monocytes, CTL and cDCs) (**Supplementary Figure 1A**). Our study examined 23 key genes (see method) involved in the m^6^A modification process in AML cells. There is no evidence of cell-specific expression for these genes (**Supplementary Figure 1B**). We used AUCell to score m6A sets within individual cells to further understand m^6^A activity. The AUC values across all cells showed two peaks, with 8465 cells showing relatively higher AUC values when the AUC threshold was set to 0.048 (**Figure 1A**). Next, we categorized AML cells into two distinct groups based on their m^6^A modification: m^6^A active (AUC value > 0.048) vs. m^6^A inactive (AUC value ≤ 0.048). We performed differential gene analysis on cells with varying m^6^A activity and observed 59 significantly upregulated genes and 46 significantly downregulated genes (|log2FC| > 0.25 and p-value < 0.01, **Figure 1B**). Furthermore, we performed pathway enrichment analysis on these genes, revealing their impact on myeloid leukocyte cytokine production, mRNA metabolism, and immune effector functions (**Figure 1C**). We found that the Hallmark gene set, which is comprised of a broad range of biological processes, provided a more comprehensive representation of differences between m^6^A-active and m^6^A-inactive cells (**Figure 1D**). Finally, we found that a higher proportion of leukemic cells (χ^2^ = 88.81, p-value = 4.34e−21, **Supplementary Figure 1C**), such as LSPCs, Mono-like, ProMono-like, GMP-like and cDC-like blasts were identified as m^6^A-active cells than immune cells (χ^2^ = 422.95, p-value = 8.17e−84, **Figure 1E**). This suggests that m^6^A modification activity may play a critical role in regulating a wide range of biological processes and underscores the importance of considering the overall functional landscape of cellular processes when studying the effects of m^6^A modification.

### 3.2 Effects of m^6^A modification status on gene regulatory networks and biological processes in leukemic cells

**Figure 2:**
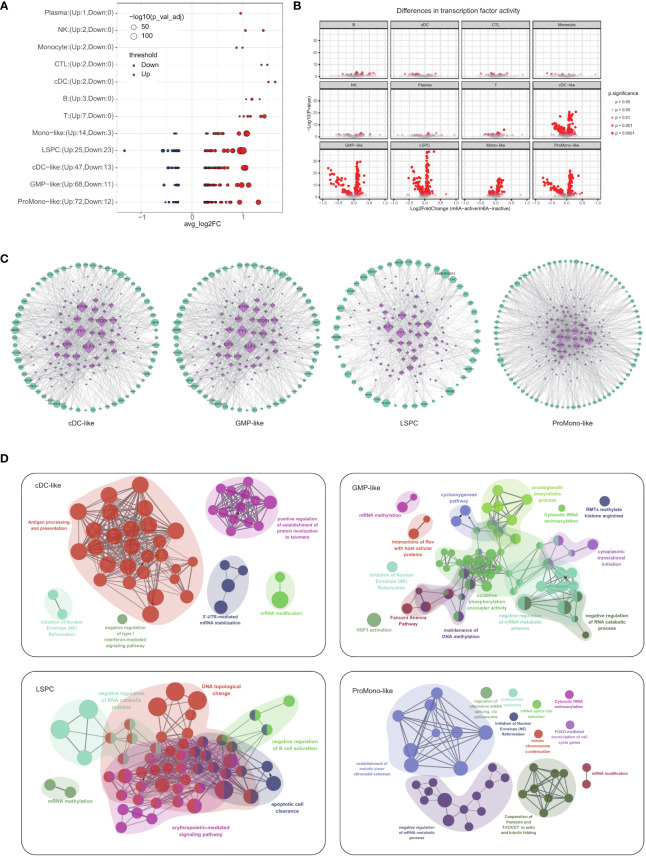
Effect of m^6^A modification status on transcriptional regulatory networks and pathway enrichment in four types of leukemia cells **(A)** Bubble plot showing the genes that were differentially expressed between m^6^A-active and m^6^A-inactive in each cell type. Node sizes correspond to -log10 of FDR adjusted p-value. **(B)** The volcano plots of differential transcription factor activity analysis of single-cell RNA sequencing data performed on each cell type comparing m^6^A-active cells vs. m^6^A-inactive cells. **(C)** Regulatory networks of transcription factors in four major types of leukemia cells (cDC-like, GMP-like, LSPCs and ProMono-like). **(D)** Pathway enrichment network of four major types of leukemia cells.

We identified leukemic cells, including LSPCs, cDC-like, GMP-like, Mono-like and ProMono-like blasts, which exhibited the most significant impact on m^6^A modification, as evidenced by the observation of more up- and down-regulated genes in their active and inactive states compared to other cell types (**Figure 2A**). To fully appreciate the complexity of m^6^A modification, it is essential to complement gene expression analysis with an understanding of the underlying gene regulatory network. In this regard, we have constructed regulatory networks for leukemic and immune cells with distinct m^6^A modification patterns using SCENIC, a transcription factor-based gene regulatory network. Compared with immune cells, leukemia cells showed greater differences in transcription factor activity between m^6^A-active and m^6^A-inactive cells (**Figure 2B**). Some transcription factors have higher activity in leukemic cells with active m^6^A modification, such as *BATF, GABPA, E2F8, E2F2, E2F3, E2F7, ELF1, YY1, HOXA9* (30–35) (**Supplementary Figure 1D**, **Supplementary Table 1**). The transcription factors *ELF1, YY1, and E2F* are key regulatory elements with a ubiquitous impact across diverse cell types, exerting a significant influence on the modulation of gene expression changes associated with M^6^A modifications (**Figure 2C**; **Supplementary Figures 1D, E**). Tumor necrosis factor α-inducible protein 8 (*TNFAIP8*) is a novel anti-apoptotic molecule that plays a role in AML chemoresistance (31, 36). *ELF1* was reported to be responsible for the upregulation of *TNFAIP8* expression in human AML patients (31). *YY1* has been reported to bind to the promoter region of *METTL3* and promote its expression, resulting in increased AML cell proliferation (34). E2F transcription factor 1 (*E2F1*) was reported to be involved in AML cell differentiation recently (35).

To identify the unique m^6^A-related genes in each of these cell types, we developed a three-step workflow (**Supplementary Figure 1F**). Firstly, we obtained the marker genes for each cell type, which enabled us to accurately classify the cells. Secondly, we performed differential expression analysis between m^6^A active and inactive cells in each cell type. Finally, we obtained a list of unique m^6^A-related genes for each cell type by overlapping the two sets of genes. We performed pathway enrichment analysis of the identified m^6^A-related genes for each cell type, which revealed their biological significance. In cDC-like blasts, m^6^A modification affected pathways related to antigen processing and presentation and 3’-UTR-mediated mRNA stabilization (**Figure 2D**). In GMP-like blasts, m^6^A modification affected pathways related to negative regulation of mRNA metabolic process and maintenance of DNA methylation (**Figure 2D**). In LSPCs, m^6^A modification affected pathways related to DNA topological change and mRNA methylation (**Figure 2D**). In ProMono-like blasts, m^6^A modification impacted pathways related to negative regulation of mRNA metabolic process and mRNA modification (**Figure 2D**). In Mono-like blasts, m^6^A modification affected pathways related to RNA metabolic process and transport (**Supplementary Figure 1G**). These findings provide insights into the distinct roles of m^6^A modification in different cell types and highlight the importance of this epigenetic mechanism in regulating cellular function and its relationship to AML progression.

### 3.3 The impact of m^6^A modification on gene regulatory networks in AML

**Figure 3:**
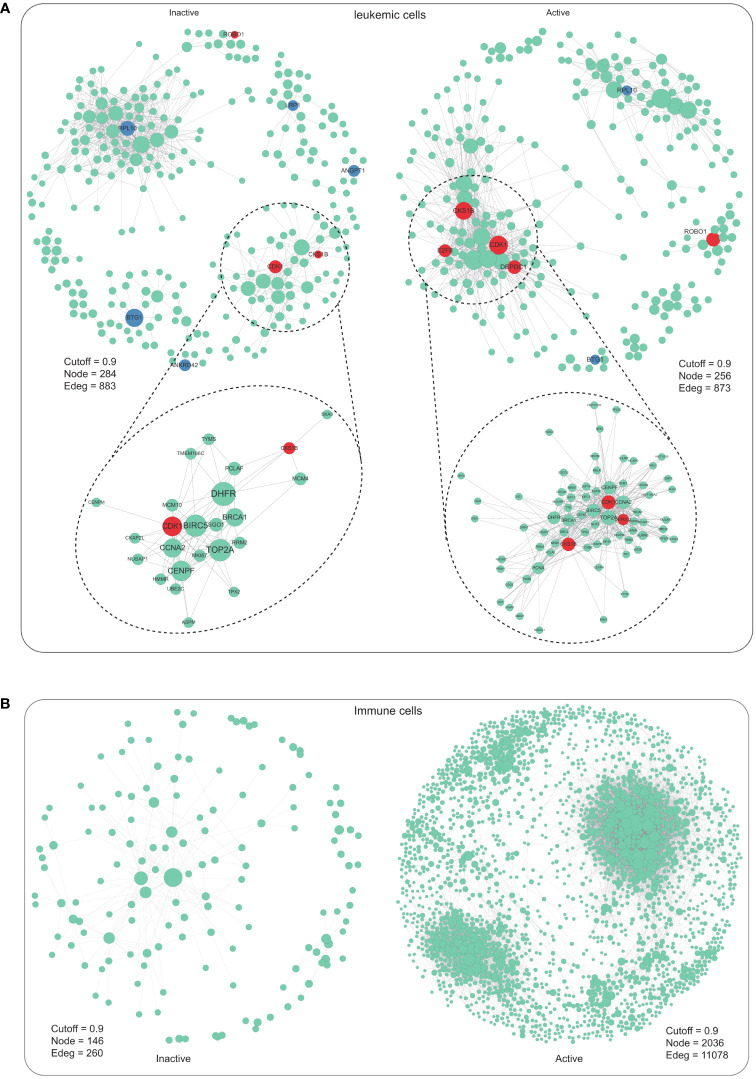
Gene regulatory network of m^6^A-inactive and m^6^A-active cells in leukemic cells and immune cells. **(A)** Gene regulatory network of m^6^A-inactive and m^6^A-active cells in leukemic cells (inactive: Cutoff = 0.9, Node = 284, Edge = 883; active: Cutoff = 0.9, Node = 256, Edge = 873). **(B)** Gene regulatory network of m^6^A-inactive and m^6^A-active cells in immune cells (inactive: Cutoff = 0.9, Node = 146, Edge = 260; active: Cutoff = 0.9, Node = 2036, Edge = 11078).

To better understand how leukemia cell or immune cell global regulatory networks are altered in m^6^A modification pattern, a gene regulatory network (GRN) reasoning methodology called bigSCale(25, 26) was implemented to m^6^A-active or m^6^A-inactive cells (**Supplementary Table 2**). In leukemic cells, the m^6^A-active and m^6^A-inactive GRN exhibited the similar densities (**Figure 3A**). To assess the biological relevance of the differences between m^6^A-active and m^6^A-inactive networks in leukemic cells, the major distinguishing variables PageRank delineating their topology were investigated. We distinguished the top 5 influencer genes ranked by PageRank, as a proxy for a gene’s influence on the network. Many of these genes have been previously linked to AML pathology, such as *CDK1* and *CKS1B* (37, 38) (**Figure 3A**). As an illustration, two genes, *CDK1* and *CKS1B*, known for their involvement in G1/S and G2/M phase transitions of the eukaryotic cell cycle, exhibited no significant differential expression (p-value > 0.05) but concurrently showed increased centrality measures (as depicted in **Figure 3A**). This finding is particularly intriguing, given that leukemic cells, despite being the most deregulated cell type in the initial AML analysis, did not originally highlight the significance of *CDK1* and *CKS1B* in this context. In each LPSCs, cell cycle distribution was determined by Zeng et al. (20). Cycling LSPCs exhibited greater m^6^A modification activity (**Supplementary Figure 1H**). However, in immune cells, the topology of the m^6^A-active GRN showed a relatively higher density in active cells compared with inactive cells, as reflected by gene-gene relationships and modularity (**Figure 3B**). Overall, our findings suggest that m^6^A modification pattern in leukemia cells or immune cells may have different effects on GRN topology, which could provide important insights into the pathological mechanisms of diseases.

### 3.4 Classification of AML subtypes using the m^6^A-related genes in leukemia cells

**Figure 4:**
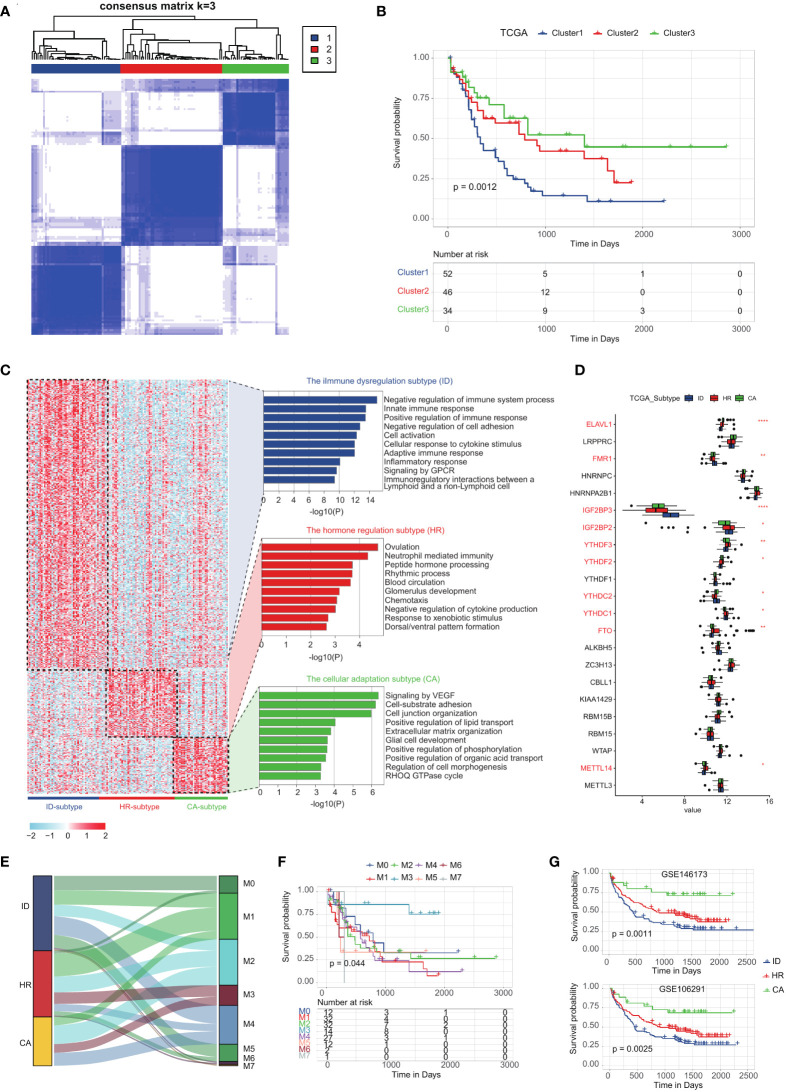
Classification of AML subtypes using the m^6^A-related genes in leukemia cells by K-means analysis. **(A)** K = 3 was identified as the optimal value for consensus clustering. **(B)** Kaplan-Meier curves of overall survival (OS) among the three subtypes in the TCGA LAML cohort. **(C)** The enrichment statistics of ssGSEA signaling pathways of immune dysregulation subtype, hormone regulation subtype and cellular adaptation subtype. **(D)** The expression levels of m^6^A-associated genes of immune dysregulation subtype, hormone regulation subtype and cellular adaptation subtype. **(E)** Sankey plot showing the correlation between the classification of AML subtypes using the m^6^A-related genes and French-American-British classification of acute myeloid leukemia. **(F)** The survival curve demonstrates the prognostic outcomes of FAB classification from TCGA-LAML. **(G)** Kaplan-Meier curves of OS among the three subtypes in two other GEO datasets (GSE146173 and GSE106291).

We integrated the unique m^6^A-related genes of cDC-like blasts, GMP-like blasts, LSPCs, Mono-like blasts and ProMono-like blasts, and performed a consensus clustering analysis on TCGA LAML samples (**Supplementary Table 3**, see method). Our clustering analysis revealed three distinct subtypes with high consistency and robustness, which were identified based on the cumulative distribution function (CDF) of the consensus matrix (**Figure 4A**). Subsequently, we conducted survival analysis on these subtypes using Kaplan-Meier curves and found significant differences in OS between the subtypes (**Figure 4B**). We performed differential gene expression analysis on each subtype and identified pathways that were significantly enriched (**Figure 4C**). The first subtype, characterized by the poorest survival, demonstrated significant enrichment in immune system processes such as negative regulation of immune system processes, innate immune response, and adaptive immune response, in addition to pathways related to cell activation, cytokine stimulus, and inflammatory response. Based on these findings, we defined this subtype as the immune dysregulation (ID) subtype (**Figure 4C**). The second subtype, with intermediate survival, exhibited significant enrichment in pathways related to ovulation, response to xenobiotic stimulus, and neutrophil-mediated immunity, along with pathways related to chemotaxis, blood circulation, and hormone processing. We defined this subtype as the hormone regulation (HR) subtype (**Figure 4C**). The third subtype, which had the best survival, demonstrated significant enrichment in pathways related to cell morphogenesis, extracellular matrix organization, and lipid transport, as well as pathways related to cell junction organization and signaling by VEGF. Based on these findings, we have defined a new subtype, called the cellular adaptation (CA) subtype (**Figure 4C**; **Supplementary Table 4**).

To comprehensively characterize the landscape of m^6^A modifications across subtypes, we have examined the expression levels of m^6^A-associated genes. Our analysis revealed that each subtype exhibits a distinctive m^6^A expression profile, suggestive of subtype-specific regulation of m^6^A modification (**Figure 4D**). For example, the immune dysregulation subtype was characterized by high expression of *IGF2BP3*, *IGF2BP2*, and *FMR1*, and low expression of *ELAVL1*, *FTO*, and *METTL14* (**Figure 4D**). We further investigated the correlation between AML subtype classification using m^6^A-related genes and the French-American-British (FAB) classification AML (39). The classification obtained from m^6^A-related genes, although not specifically correlated with FAB classifications, demonstrates a robust prognostic diagnostic capability (**Figures 4E, F**). The three subtypes are characterized from different aspects, including m^6^A gene expression, m^6^A modification activity of leukemic cells, and subtype distribution. To examine the robustness of our subtype identification, we have developed a composite machine-learning classifier based on m^6^A-related genes and the DEGs among the three subtypes (see method, **Supplementary Table 5**). We further validated the stability and reproducibility of the three subtypes in two additional GEO datasets, providing strong evidence of their statistical robustness (**Figure 4G**; **Supplementary Figure 2**; **Supplementary Tables 6**, **7**).

### 3.5 Comparison of genomic alterations of the three AML subtypes

**Figure 5:**
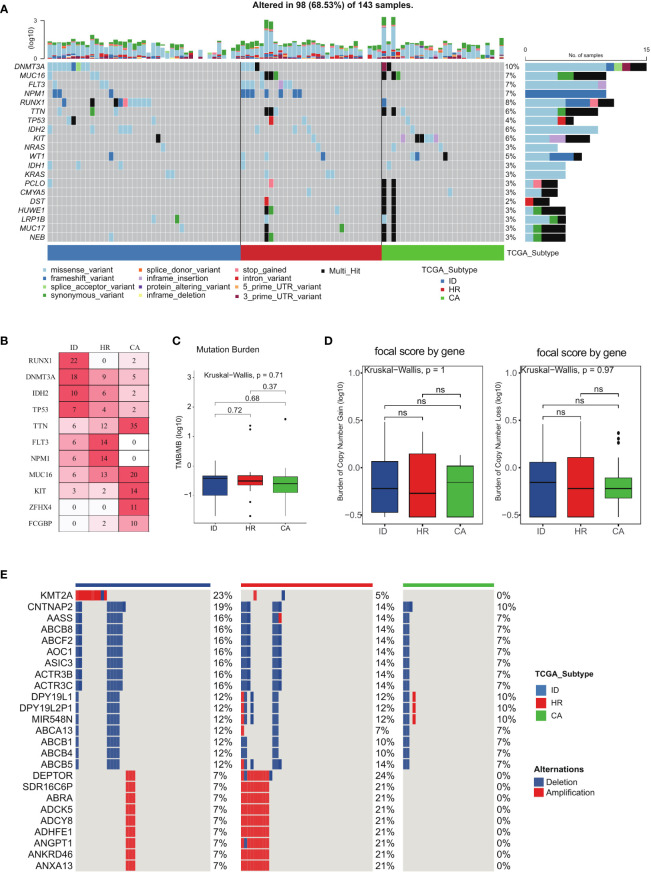
Comparison of genomic alterations of the three AML subtypes. **(A)** The genomic alterations among the three subtypes of the TCGA LAML cohort. **(B)** Mutation percentage of mostly mutated genes. **(C)** Tumor mutant burden difference among the three subtypes in the TCGA LAML cohort. **(D)** The Burden of Copy Number gain and the Burden of Copy Number Loss among the three subtypes in the TCGA LAML cohort. **(E)** Distinct Copy number alterations (CNA) profile among the three subtypes.

We utilized oncoplot to assess the genomic alterations in all subtypes of the TCGA LAML cohort (**Figures 5A, B**). Our analysis revealed that *RUNX1*, *DNMT3A*, and *IDH2* mutations were most frequently observed in the immune dysregulation subtype, while being least prevalent in the other two subtypes (**Figure 5B**). Additionally, *TTN, MUC16, KIT, ZFHX4*, and *FCGBP* mutations were mainly observed in the cellular adaptation subtype (**Figure 5B**). Although the TMB of the immune dysregulation subtype was found to be higher than the remaining two subtypes, the difference was not statistically significant (**Figure 5C**). Similarly, the Burden of Copy Number gain was higher in the cellular adaptation subtype compared to the other two subtypes, whereas the Burden of Copy Number Loss was higher in the immune dysregulation subtype than in the other two subtypes; however, these differences failed to achieve statistical significance (**Figure 5D**). Notably, the copy number variation in *KMT2A* was specifically present in the immune dysregulation subtype (**Figure 5E**), highlighting the potential role of this gene in driving immune dysregulation in AML.

### 3.6 The three AML subtypes exhibited different immune statuses

**Figure 6:**
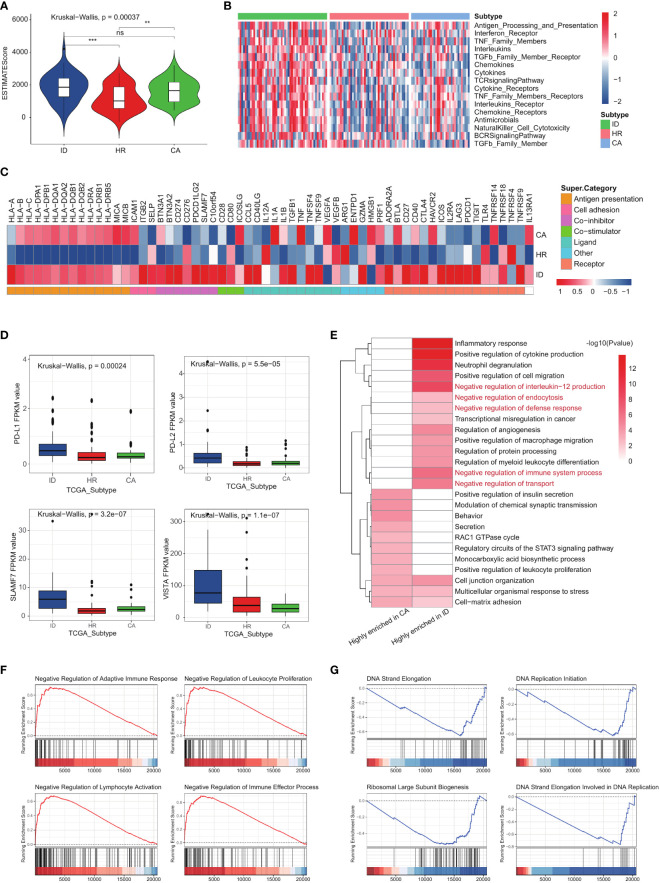
The three AML subtypes exhibited different immune statuses. **(A)** The ESTIMATE scores among the three subtypes in the TCGA LAML cohort. **(B)** The activation degree of 17 immune pathways in each tumor sample among the three subtypes. **(C)** The expression levels of 78 immunomodulators among the three subtypes. **(D)** Boxplot showing the activation status of suppressor proteins in different subtypes. **(E)** The pathway enrichment analysis on the top 100 differentially expressed genes in CA and ID subgroup. **(F, G)** GSEA results showing the activated signaling pathways in ID and CA subgroup. p-value > 0.05: ns, p-value < 0.05:*, 0.05 < p-value < 0.01: **, 0.01<p-value<0.001:***.

The immune differences in the three subtypes of AML were explored through a comprehensive analysis of the immune microenvironment. We employed the ESTIMATE algorithm to calculate immune scores, revealing that patients in the immune dysregulation subtype exhibited significantly higher ESTIMATE scores compared to those in the other two subtypes (**Figure 6A**). However, the prognosis of the cellular adaptation subtype was found to be better than that of the other two subtypes, despite its higher immune score. Further, the activation states of immune-related pathways also were measured (**Figure 6B**). Several pathways crucial for immune function activation, including ‘Antigen processing and presentation,’ ‘NK cell cytotoxicity,’ and the ‘TCR signaling pathway,’ consistently exhibited upregulation in both the immune dysregulation subtype and the cellular adaptation subtype. Consistent with the ESTIMATE algorithm, all immune pathways within the tumor microenvironment get the highest activation level in the immune dysregulation subtype. We conducted a more detailed examination of the expression levels of 78 immunomodulators in each subtype (as shown in **Figure 6C**). Notably, the majority of differentially expressed immunomodulators exhibited the highest levels in both the immune dysregulation subtype and the cellular adaptation subtype. This includes key genes related to antigen presentation (*HLA-B, HLA-C, HLA-DPA1, HLA-DPB1, HLA-DQA1, HLA-DQA2, HLA-DQB1, HLA-DQB2, HLA-DRA, HLA-DRB1, HLA-DRB5, MICA* and *MICB*). The total expressions of 78 immunomodulators were also the highest in the immune dysregulation subtype. However, it must be emphasized that unlike the cellular adaptation subtype, the suppressor proteins, such as *CD274, CD276, PDCD1LG2, BTN3A1, BTN3A2, SLAMF7* and *C10orf54* in the immune dysregulation subtype are also activated (**Figures 6C, D**; **Supplementary Figure 3A**). This is particularly remarkable because there was a significant difference in survival risk between the two subtypes (**Figures 4B, F**), but the immune dysregulation subtype and the cell adaptation subtype exhibits highly active immune functions. This compelled us to further analyze the key differences that determine the survival risk in these two subtypes and conducted pathway enrichment analysis on the top 100 differentially expressed genes in each subgroup. As expected, the immune dysregulation subtype not only exhibited highly activated immune responses, but also highly enriched pathways involved in negative regulation of immune responses (**Figure 6E**). Further GSEA confirmed our findings: in the immune dysregulation subtype, immune response and immune response processes were negatively regulated (**Figure 6F**), while the cell adaptation subtype exhibited enrichment in pathways related to DNA synthesis and protein synthesis (**Figure 6G**).

As a result, the clinical prognosis and biological characteristics of each subtype in two GEO cohorts closely resembled those of the corresponding subtypes identified in the TCGA LAML cohort (see **Supplementary Figures 3A, B**). Furthermore, akin to the TCGA LAML cohort, the levels of immune pathway activation and expression of immunomodulators were highest in the immune dysregulation subtype, reinforcing the robust and reliable nature of our identification of immune features among the three LAML subtypes (see **Supplementary Figures 3A, B**).

### 3.7 The three AML subtypes exhibited different drug resistance

**Figure 7:**
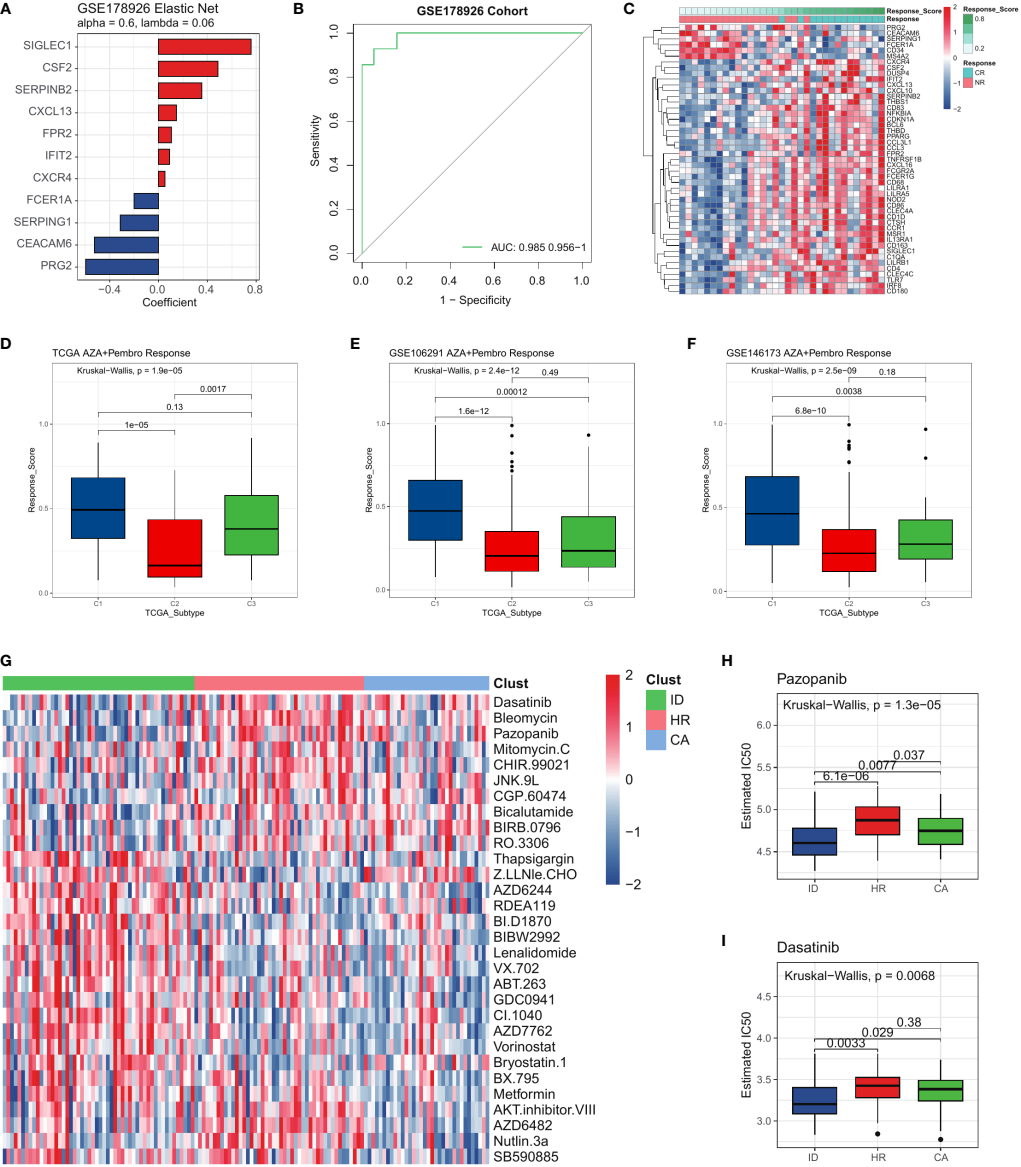
The three AML subtypes exhibited different drug resistance. **(A)** Features selected by Elastic-Net regression to differentiate between CR and NR samples. **(B)** ROC curves for the performance of GSE178926 cohort in predicting immunotherapy response. **(C)** Differentially expressed genes between CR and NR samples in GSE178926 cohort (Response score is the immunotherapy response score calculated by the elastic network model). **(D)** Boxplot showing the AZA+Pembro response score of the TCGA LAML cohort and **(E, F)** two GEO cohorts. **(G)** The heatmap showing the sensitivity of the three AML subtypes to different compounds. **(H, I)** Sensitivity of the three AML subtypes to Pazopanib, Dasatinib.

To further investigate whether the immune dysregulation subtype had a better response rate to immune checkpoint blockade, we conducted a reprofiling of primary bone marrow (BM) samples obtained from 33 adult patients diagnosed with either newly diagnosed or relapsed/refractory AML who underwent treatment with AZA+Pembro (Azacitidine in Combination with Pembrolizumab, ClinicalTrials.gov NCT02845297). We investigated differentially expressed genes (DEGs) at the baseline between patients who eventually achieved Complete Remission (CR) and those who did not respond (NR). To precisely delineate immunotherapy responses distinguishing CR and NR samples in an unbiased manner, we conducted further refinement of the gene list between CR and NR samples. We employed Elastic Net penalized logistic regression (as shown in **Figure 7A**) to ensure the inclusion of only the most significant genes in the model, while also accounting for the high degree of correlation observed among certain immunotherapy responses. Collapsing our immunotherapy signature into a gene-weighted response score, we confirmed its ability to distinguish CR from NR samples through ROC analysis in the cohort (**Figures 7B, C**). Fortunately, the immune dysregulation subtype is more sensitive to AZA+Pembro therapy compared to other subtypes. (**Figure 7D**). This conclusion was validated in the other two publicly available GEO datasets, confirming that the immune dysregulation subtype is more sensitive to AZA+Pembro therapy compared to other subtypes (**Figures 7E, F**). Further, we employed molecular subtyping to stratify patients for precision medicine. To assess whether the three AML subtypes exhibited varying sensitivities to different drug profiles, we utilized the Genomics of Drug Sensitivity in Cancer (GDSC) database. Our findings revealed that different AML subtypes displayed sensitivity to distinct compounds, reinforcing the significance of molecular subtyping. (**Figure 7G**). We found that the Immune dysregulation patients were more sensitive to Pazopanib and Dasatinib (**Figures 7H, I**), and more tolerance to Vorinostat, Metformin, AZD6244 and Nutlin-3a etc. (**Figure 7G**). To investigate whether the drug sensitive result of the three subtypes of AML were repeatable in other datasets, we did a similar analysis on two GEO cohorts. As a result, the chemotherapy response of each subtype in two GEO cohorts were the same as the corresponding subtype identified in the TCGA LAML cohort (**Supplementary Figure 4**, **Supplementary Tables 8**, **9**).

## 4 Discussion

Numerous investigations have firmly established the critical role of m^6^A modification in the etiology of AML and have identified singular molecular subtypes of AML based on changes in the m^6^A modifying enzymes (40, 41). Despite these advances, most of these studies focused on the bulk tissues, while largely neglecting the relevance of the cell hierarchy to AML initiation and progression(42). Neglecting the heterogeneity of m^6^A modification at the cell level may impair our understanding of fundamental mechanisms underlying AML, given that cellular properties significantly shape the microenvironment and ultimately determine the disease outcome (18, 20). In order to bridge this gap in knowledge, we undertook this investigation to incorporate information on m^6^A modification status and cell-level features in AML with prudence.

Several methods, including principal component analysis (PCA), single-sample gene set enrichment analysis (ssGSEA), and gene set variation analysis (43) (GSVA) are commonly used in bulk RNA-seq analysis to define the activation level of biological processes. These methods have also been applied extensively to investigate m^6^A modification activity in AML patients (44, 45). However, due to the significant sparsity issues in single-cell sequencing data, these conventional methods may not be well-suited for single-cell data analysis (46). Additionally, the 23 m^6^A modifying enzymes differ from conventional biological process gene sets as they consist of three types of enzymes: writers, readers and erasers, each performing a distinct function (47). These enzymes can form numerous combinations to participate in the m^6^A modification process in cells (48). Therefore, we used AUCell (22) to determine the m^6^A modification activity of each cell, taking into account the characteristics of single-cell data and the properties of m^6^A modifying enzymes. We categorized each cell into an active or inactive group based on its m^6^A modification activity. Notably, our results showed that m^6^A modification was more active in AML cells than in non-leukemic immune cells, highlighting the potential role of m^6^A modification in AML pathogenesis.

Transcription factor regulatory networks within cells are frequently constructed, offering an exciting opportunity for high-resolution identification of distinct transcriptional states and transitions, such as the m^6^A modification status (22). In a recent study, Zeng et al. employed the SCENIC network to delineate a subset of cells characterized by activation of *CDK6* and *E2F3*, subsequently categorizing them as leukemia cells associated with cell cycle initiation (20). Subsequently, Guo et al. conducted VIPER to elucidate the transcription factor (TF) activity in AML progenitor cells (44). Our investigation aimed to elucidate the impact of differential m^6^A activity on regulatory networks. Notably, our findings demonstrated that m^6^A modifications exert a more pronounced effect on the transcription factor regulatory network within leukemia cells, with transcription factors in m^6^A-active leukemia cells exhibiting more conspicuous transcriptional regulatory activity. However, it is noteworthy that there is no significant correlation between transcription factor regulatory activity and m^6^A modification activity in immune cells. Specifically, TFs such as *YY1, E2F1*, and *E2F8* were found to be closely associated with m^6^A activity within leukemia cells. Extensive evidence supports the role of *YY1* and *E2F1* in the regulation of cell differentiation and proliferation (34, 35). Additionally, we considered the topological changes in gene regulatory networks, which may reflect the complexity of intracellular gene expression(26). Importantly, AML cells with active m^6^A modifications and those with inactive m^6^A modifications exhibited similar gene-gene interaction densities. In contrast, gene interaction networks in non-AML immune cells were significantly influenced by m^6^A modifications. This suggests that m^6^A modifications in AML cells may impair their ability to coordinate gene networks effectively.

Numerous previous studies have established the significant influence of m^6^A modification on the prognosis of patients with AML(48). Nevertheless, the precise mechanism underlying this effect has not been thoroughly investigated. In this study, we present an innovative hypothesis suggesting that m^6^A modification primarily affects leukemic cells, thereby altering the prognostic status of AML patients. Our analysis revealed the existence of a high-risk subtype of AML termed “immune dysregulated subtype,” which exhibits *RUNX1* mutations and *KMT2A* copy number variations (49, 50). Notably, our findings shed light on AML molecular pathogenesis and may offer crucial implications for personalized therapeutic strategies. To this end, we aim to determine the altered immune status and sensitivity to immunotherapy of this high-risk subtype. After identifying this high-risk subtype, our goal is to determine its altered immune status and sensitivity to immunotherapy. It is important to note that this subtype is active in the immune system and has shown sensitivity to immunotherapy. Our analysis revealed that Pazopanib and Dasatinib are highly sensitive to this subtype, and potential therapies for treating AML using these drugs have been reported in the literature (49, 51). This information on immunotherapy can be used to support clinical treatment and diagnosis.

Our study was the first to demonstrate the m^6^A modification information in AML at the single-cell level. We found that the m^6^A modification status of leukemic cells are strongly correlated with patient prognosis. By combining m^6^A modification information and cellular information, we identified a high-risk subtype and elucidated its clinical features. Furthermore, our study developed a high-precision machine learning composite model that integrates multiple mainstream machine learning algorithms. The robustness of our findings has been validated through one-by-one confirmation in a mainstream cohort. Nevertheless, we do acknowledge certain limitations of our study. In our study, we employed AUCell to assess the activation status of m^6^A-related genes based on their expression rank. This approach allowed us to form sets of genes that could potentially act in coordination to mediate m^6^A modifications. However, it is important to note that m^6^A enzymes often function in a collaborative and interdependent manner, and pinpointing precise coordination mechanisms can be challenging (48). Additionally, distinguishing between immune cells and leukemia cells in bulk RNA-seq data can be challenging because these cell types may share common biological processes or differential gene expression profiles (20).

## Data availability statement

The original contributions presented in the study are included in the article/**Supplementary Material**. Further inquiries can be directed to the corresponding authors.

## Ethics statement

Ethical approval was not required for the study involving humans in accordance with the local legislation and institutional requirements. Written informed consent to participate in this study was not required from the participants or the participants’ legal guardians/next of kin in accordance with the national legislation and the institutional requirements.

## Author contributions

ZL: Conceptualization, Data curation, Formal Analysis, Methodology, Software, Writing – original draft, Writing – review & editing. XL: Data curation, Formal Analysis, Investigation, Methodology, Writing – original draft, Writing – review & editing. LW: Funding acquisition, Supervision, Writing – review & editing. HZ: Data curation, Investigation, Visualization, Writing – original draft. SW: Visualization, Writing – original draft. GY: Funding acquisition, Project administration, Supervision, Writing – review & editing. DW: Funding acquisition, Project administration, Supervision, Writing – review & editing. JC: Conceptualization, Funding acquisition, Project administration, Supervision, Writing – review & editing. JH: Conceptualization, Funding acquisition, Project administration, Supervision, Writing – review & editing.

## Supplementary material

Supplementary Figure 1 Effect of m^6^A modification status on active and inactive m^6^A methylated cells in AML. **(A)** t-SNE visualization of 13,653 single-cell transcriptomes (points), with similar cells positioned closer together. Cell type annotation were obtained from Zeng et al. (20). **(B)** Heatmap showing the expression of 23 selected cell-type-specific genes (rows) across 13,653 single cells ordered by cell-type annotations as shown in A (columns). **(C)** The stacked bar chart displays the proportions of m^6^A modification-active and inactive cells in leukemia and immune cells. **(D)** Line plots show the number of cell types of transcription factors with significantly different activity between m^6^A-active cells and m^6^A-inactive cells in immune cells (blue) and leukemic cells (orange). **(E)** The network plot shows the regulatory relationship between transcription factors and differentially expressed genes (m^6^A-active cells vs. m^6^A-inactive cells) in Mono-like blasts. **(F)** The workflow shows the screening process of cell-specific m^6^A-related genes. **(G)** The network plot shows the pathways the pathways enriched by Mono-like blasts-specific m^6^A-related genes. **(H)** Boxplot shows the m^6^A modification activity of LSPCs in the three states (Quiescent, Primed, Cycle).

Supplementary Figure 2 The Robustness of composite machine learning classification models for the identification of subtypes in the GEO dataset. Kaplan-Meier curves of overall survival (OS) among the three subtypes classified by different machine learning models in the GSE106291 cohort (up) and the GSE146173 cohort (down).

Supplementary Figure 3 Identification of immune features of the three AML subtypes in the GEO dataset. **(A)** The ESTIMATE scores and the activation degree of 17 immune pathways among the three subtypes in the GSE106291 cohort and GSE146173 cohort. **(B)** The expression levels of 78 immunomodulators among the three subtypes in the GSE106291 cohort and GSE146173 cohort.

Supplementary Figure 4 The sensitivity of the three AML subtypes to different compounds in the GEO dataset. **(A)** The heatmap showing the sensitivity of the three AML subtypes to different compounds (GSE106291). **(B)** Sensitivity of the three AML subtypes to Pazopanib, Dasatinib (GSE106291). **(C)** The heatmap showing the sensitivity of the three AML subtypes to different compounds (GSE146173). **(D)** Sensitivity of the three AML subtypes to Pazopanib, Dasatinib (GSE146173).

Supplementary Table 1 Results of differential transcription factor activity analysis.

Supplementary Table 2 Results of BigScale2-based gene regulatory network analysis.

Supplementary Table 3 Cell-specific m^6^A-associated gene set.

Supplementary Table 4 The classification information of the TCGA.

Supplementary Table 5 Gene set used to construct models that identify molecular subtypes.

Supplementary Table 6 Accuracy statistics for multiple machine learning models.

Supplementary Table 7 The classification information of the validation set.

Supplementary Table 8 Results of drug susceptibility prediction.

Supplementary Table 9 IC50 range table for each cell line corresponding to the drug.

Supplementary File 1 Count matrix of AML patients from TCGA database.

Supplementary File 2 Count matrix of AML patients from GSE106291 database.

Supplementary File 3 Count matrix of AML patients from GSE146173 database.
