# Supplementary figures and images for: Integrated analysis of single-cell RNA-seq and bulk RNA-seq reveals RNA N6-methyladenosine modification associated with prognosis and drug resistance in acute myeloid leukemia
Source: Front Immunol. 2023 Oct 31;14:1281687. doi: 10.3389/fimmu.2023.1281687 (PMC10644381; doi:10.3389/fimmu.2023.1281687)

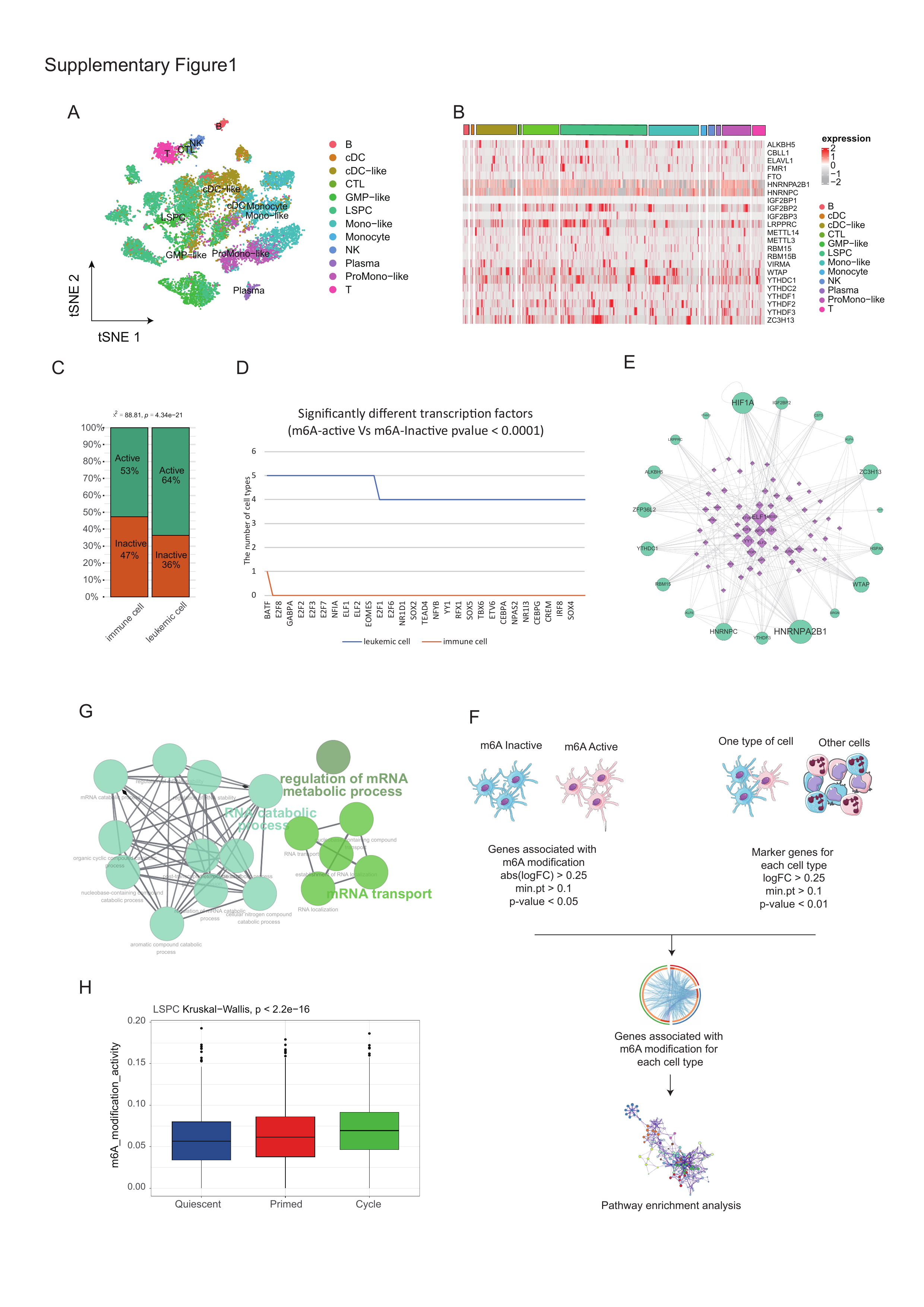

Supplement: Supplementary Figure 1 — Effect of m6A modification status on active and inactive m6A methylated cells in AML. (A) t-SNE visualization of 13,653 single-cell transcriptomes (points), with similar cells positioned closer together. Cell type annotation were obtained from Zeng et al. (20). (B) Heatmap showing the expression of 23 selected cell-type-specific genes (rows) across 13,653 single cells ordered by cell-type annotations as shown in A (columns). (C) The stacked bar chart displays the proportions of m6A modification-active and inactive cells in leukemia and immune cells. (D) Line plots show the number of cell types of transcription factors with significantly different activity between m6A-active cells and m6A-inactive cells in immune cells (blue) and leukemic cells (orange). (E) The network plot shows the regulatory relationship between transcription factors and differentially expressed genes (m6A-active cells vs. m6A-inactive cells) in Mono-like blasts. (F) The workflow shows the screening process of cell-specific m6A-related genes. (G) The network plot shows the pathways the pathways enriched by Mono-like blasts-specific m6A-related genes. (H) Boxplot shows the m6A modification activity of LSPCs in the three states (Quiescent, Primed, Cycle). [file Image_1.tiff]

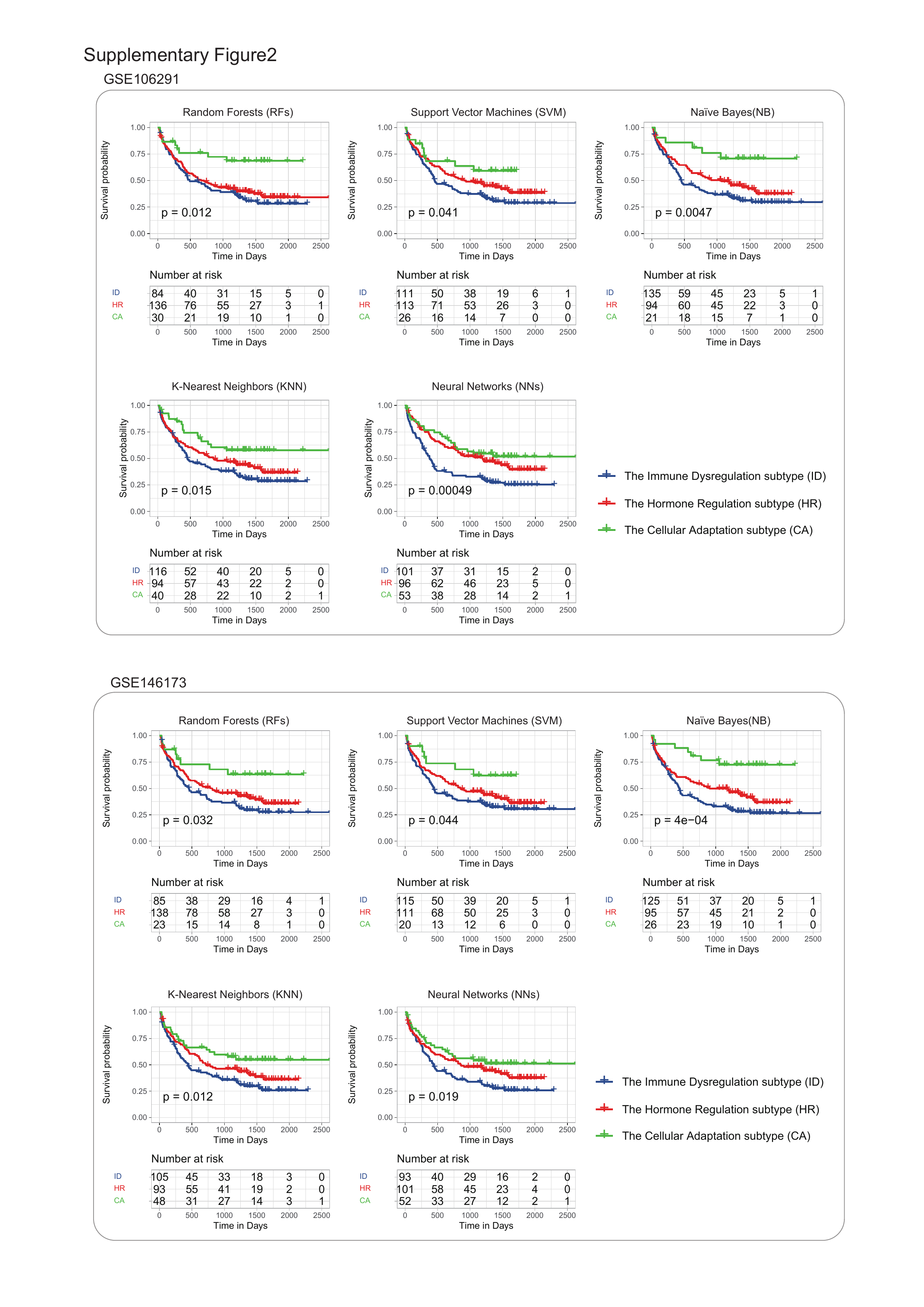

Supplement: Supplementary Figure 2 — The Robustness of composite machine learning classification models for the identification of subtypes in the GEO dataset. Kaplan-Meier curves of overall survival (OS) among the three subtypes classified by different machine learning models in the GSE106291 cohort (up) and the GSE146173 cohort (down). [file Image_2.tiff]

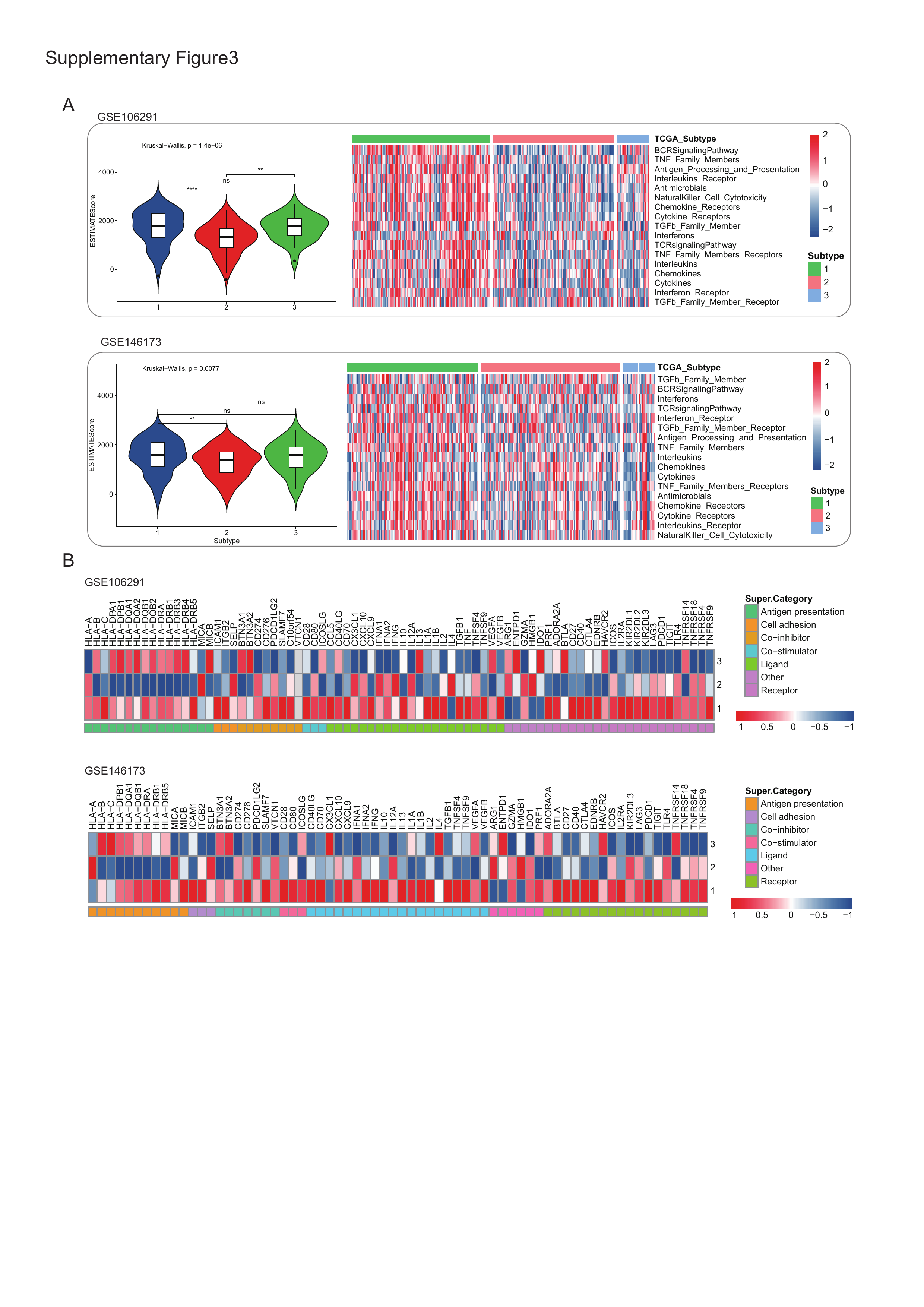

Supplement: Supplementary Figure 3 — Identification of immune features of the three AML subtypes in the GEO dataset. (A) The ESTIMATE scores and the activation degree of 17 immune pathways among the three subtypes in the GSE106291 cohort and GSE146173 cohort. (B) The expression levels of 78 immunomodulators among the three subtypes in the GSE106291 cohort and GSE146173 cohort. [file Image_3.tiff]

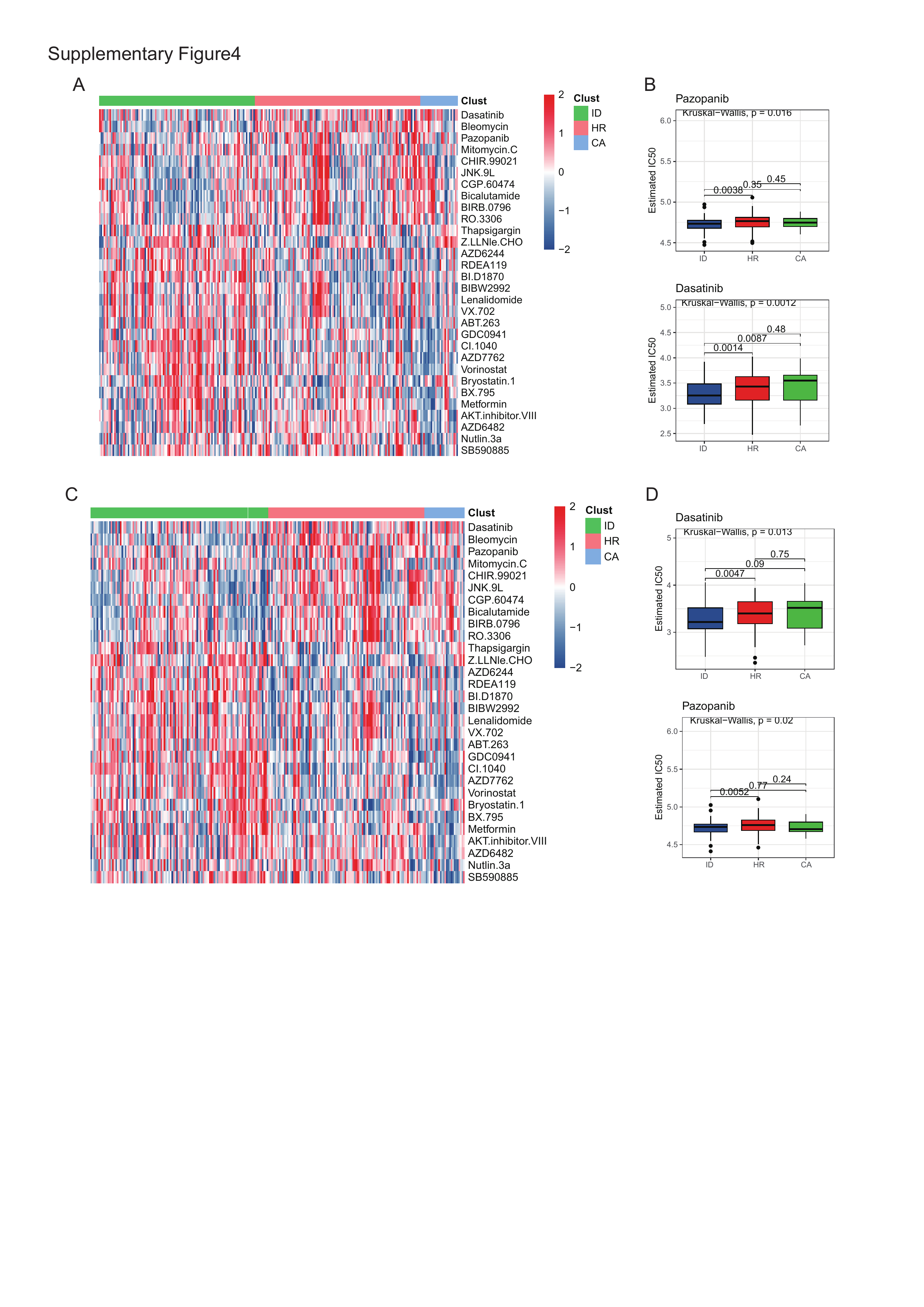

Supplement: Supplementary Figure 4 — The sensitivity of the three AML subtypes to different compounds in the GEO dataset. (A) The heatmap showing the sensitivity of the three AML subtypes to different compounds (GSE106291). (B) Sensitivity of the three AML subtypes to Pazopanib, Dasatinib (GSE106291). (C) The heatmap showing the sensitivity of the three AML subtypes to different compounds (GSE146173). (D) Sensitivity of the three AML subtypes to Pazopanib, Dasatinib (GSE146173). [file Image_4.tiff]
